# Supplementary material for: An adapted instrument to assess informed consent comprehension among youth and parents in rural western Kenya: a validation study
Source: BMJ Open. 2018 Jul 12;8(7):e021613. doi: 10.1136/bmjopen-2018-021613 (PMC6082480; doi:10.1136/bmjopen-2018-021613)
Supplement: Supplementary data [file bmjopen-2018-021613supp001.pdf]

Adolescent Ethics Research Study

Adolescents ICCA Questionnaire

The next set of questions will assess your understanding of agreement to participate in the study, including the purpose of the study, what will be expected of you, the benefits, the possible risks, and the safeguards.		
1..	Have you been told you can withdraw from this study at any time? (Choose one)	1= Yes<br>2= No<br>3= I don't know<br>8= Refuse to answer
2.	During the study, will anyone not working with KEMRI or the nearest clinic know about your health information? (Choose one)	1= Yes<br>2= No<br>3= I don't know<br>8= Refuse to answer
3.	Have you been given the name and phone number of the person to contact if you have any questions about the study? (Choose one)	1= Yes<br>2= No<br>3= I don't know<br>8= Refuse to answer
4.	Will you receive a t-shirt for taking part in the study? (Choose one)	1= Yes<br>2= No<br>3= I don't know<br>8= Refuse to answer
5.	How were participants selected into different groups in this study? (Choose one)	1= Participants were divided into different groups based on their health needs<br>2= Participants were divided into different groups equally by chance.<br>3= Participants were free to decide which group they would be placed<br>4= I don't know<br>8= Refuse to Answer
6.	At what point can you leave the study? (Choose one)	1= I can leave at any time without giving a reason<br>2= I can only leave with the permission of village elders<br>3= I can only leave when the study is over<br>4= I don't know<br>8= Refuse to Answer
7.	What does it mean when you sign the study consent form? (Choose one)	1= I would like to take part in similar studies<br>2= I do not want to take part in this study<br>3= I am agreeing to take part in this study<br>4= I don't know<br>8= Refuse to Answer
8.	If you want to join the study, but your parent/guardian does not agree, can you still join the study? (Choose one)	1= Yes, it is my choice alone<br>2= No, my parent/guardian must agree<br>3= Yes, if the researchers say that I can<br>4= I don't know<br>8= Refuse to Answer
9.	If your parent wants you to join the study, but you do not want to, are you still allowed to refuse? (Choose one)	1= Yes, it is my choice alone<br>2= No, the parents' wishes must be honored<br>3= No, the study is important for society<br>4= I don't know<br>8= Refuse to Answer

10.	What will happen if you decide to stop taking part in this study? (Choose one)	1= Nothing bad will happen, it is my choice.<br>2= This decision will affect my access to medical care in the future.<br>3= I will be fined and punished.<br>4= I don't know<br>8= Refuse to Answer
11.	Which of the following describes best why the study is being done? (Choose one)	1= To test new HIV medicines<br>2= To understand how to do HIV studies with adolescents<br>3= To check my blood for different diseases<br>4= I don't know<br>8= Refuse to Answer
12.	Which of these activities were you asked to take part in today? (Choose one)	1= Survey and HIV test<br>2= Urine sample collection<br>3= Body examination by study doctor or nurse<br>4= I don't know<br>8= Refuse to Answer
13.	Which other activities might you be invited to do? (Choose one)	1= Interviews<br>2= Testing medications<br>3= Reporting to younger adolescents how to prevent HIV<br>4= I don't know<br>8= Refuse to Answer
14.	Will you be told your HIV test results during the study? (Choose one)	1= Yes<br>2= No<br>3= I don't know<br>8= Refuse to answer
15.	If you test positive for HIV, will you be offered free treatments? (Choose one)	1= Yes, the research team will provide treatment<br>2= Yes, I will be referred to a local clinic of my choice for free treatment<br>3= No, I will not be referred to a local clinic for free treatment<br>4= I don't know<br>8= Refuse to Answer
16.	If you are invited to participate in additional interviews for this study, how will you be compensated for your participation? (Choose one)	1= A small amount of money in addition to weekly checkups<br>2= Free medicine, money, and weekly checkups<br>3= A small amount of food (oil, maize meal or sugar)<br>4= Money to cover my time for each study visit<br>8= Refuse to Answer
17.	Which describes one of the main risks involved in the study? (Choose one)	1= Becoming HIV infected<br>2= Becoming upset by my HIV test result being positive<br>3= Side effects of drugs<br>4= I don't know<br>8= Refuse to Answer

18.	Which describes the main benefit of taking part in the study? (Choose one)	1= To help other adolescents who will be involved in HIV research<br>2= Free medical care<br>3= Help with school fees<br>4= I don't know<br>8= Refuse to Answer
19.	Which one of the following best describes what makes you eligible to participate in this study? (Choose one)	1= I have not been tested in the last 6 months, have never tested positive, and am 15-17 years old<br>2= I want to know/learn my HIV status.<br>3= I was chosen by the computer<br>4= I don't know
20.	What is the difference between taking part in this study and going to the clinic for voluntary HIV testing? (Choose one)	1= There is no difference<br>2= At the clinic I would go to learn my status, but in this research I am helping researchers know how to conduct HIV research with adolescents<br>3= At the clinic you have to pay money to be tested but in this study it is free to be tested<br>4= I don't know
21.	How long will you be in this study? (Choose one)	1= For the duration of 5 years<br>2= I will be asked by the researchers to give blood one year from today<br>3= I will most likely be done with the study after today, but there is a small chance I may be asked to come back for 2 more interviews<br>4= I don't know
Thank you very much for your participation. We appreciate your help in responding to the questions. Kindly ask the research staff anything you do not understand. Do raise your hand for assistance from the research staff to exit.		

Young Adult ICCA Questionnaire

The next set of questions will assess your understanding of agreement to participate in the study, including the purpose of the study, what will be expected of you, the benefits, the possible risks, and the safeguards.		
1.	Have you been told that you can freely decide whether you will take part in this study? (Choose one)	1= Yes<br>2= No<br>3= I don't know<br>8= Refuse to answer
2.	Have you been told you can withdraw from this study at any time? (Choose one)	1= Yes<br>2= No<br>3= I don't know<br>8= Refuse to answer
3.	During the study, will anyone not working with KEMRI or the nearest clinic know about your health information? (Choose one)	1= Yes<br>2= No<br>3= I don't know<br>8= Refuse to answer

4.	Have you been given the name and phone number of the person to contact if you have any questions about the study? (Choose one)	1= Yes<br>2= No<br>3= I don't know<br>8= Refuse to answer
5.	Will you receive a t-shirt for taking part in the study? (Choose one)	1= Yes<br>2= No<br>3= I don't know<br>8= Refuse to answer
6.	How were participants selected into different groups in this study? (Choose one)	1= Participants were divided into different groups based on their health needs<br>2= Participants were divided into different groups equally by chance.<br>3= Participants were free to decide which group they would be placed<br>4= I don't know<br>8= Refuse to Answer
7.	At what point can you leave the study? (Choose one)	1= I can leave at any time without giving a reason<br>2= I can only leave with the permission of village elders<br>3= I can only leave when the study is over<br>4= I don't know<br>8= Refuse to Answer
8.	What does it mean when you sign the study consent form? (Choose one)	1= I would like to take part in similar studies<br>2= I do not want to take part in this study<br>3= I am agreeing to take part in this study<br>4= I don't know<br>8= Refuse to Answer
9.	How did you decide to join the study? (Choose one)	1= It was decided by the village leaders.<br>2= It was decided by me and it was completely voluntary<br>3= It was decided by the scientists and doctors.<br>4= It was decided by my parents<br>8= Refuse to Answer
10.	What will happen if you decide to stop taking part in this study? (Choose one)	1= Nothing bad will happen, it is my choice.<br>2= This decision will affect my access to medical care in the future.<br>3= I will be fined and punished.<br>4= I don't know<br>8= Refuse to Answer
11.	Which of the following describes best why the study is being done? (Choose one)	1= To test new HIV medicines<br>2= To understand how to do HIV studies with adolescents<br>3= To check my blood for different diseases<br>4= I don't know<br>8= Refuse to Answer
12.	Which of these activities were you asked to take part in today? (Choose one)	1= Survey and HIV test<br>2= Urine sample collection<br>3= Body examination by study doctor or nurse<br>4= I don't know

		8= Refuse to Answer
13.	Which other activities might you be invited to do? (Choose one)	1= Interviews<br>2= Testing medications<br>3= Reporting to younger adolescents how to prevent HIV<br>4= I don't know<br>8= Refuse to Answer
14.	Will you be told your HIV test results during the study? (Choose one)	1= Yes<br>2= No<br>3= I don't know<br>8= Refuse to answer
15.	If you test positive for HIV, will you be offered free treatments? (Choose one)	1= Yes, the research team will provide treatment<br>2= Yes, I will be referred to a local clinic of my choice for free treatment<br>3= No, I will not be referred to a local clinic for free treatment<br>4= I don't know<br>8= Refuse to Answer
16.	If you are invited to participate in additional interviews for this study, how will you be compensated for your participation? (Choose one)	1= A small amount of money in addition to weekly checkups<br>2= Free medicine, money, and weekly checkups<br>3= A small amount of food (oil, maize meal or sugar)<br>4= Money to cover my time for each study visit<br>8= Refuse to Answer
17.	Which describes one of the main risks involved in the study? (Choose one)	1= Becoming HIV infected<br>2= Becoming upset by my HIV test result being positive<br>3= Side effects of drugs<br>4= I don't know<br>8= Refuse to Answer
18.	Which describes the main benefit of taking part in the study? (Choose one)	1= To help other adolescents who will be involved in HIV research<br>2= Free medical care<br>3= Help with school fees<br>4= I don't know<br>8= Refuse to Answer
19.	Which one of the following best describes what makes you eligible to participate in this study? (Choose one)	1= I have not been tested in the last 6 months, have never tested positive, and am 15-19 years old<br>2= I want to know/learn my HIV status.<br>3= I was chosen by the computer<br>4= I don't know
20.	What is the difference between taking part in this study and going to the clinic for voluntary HIV testing? (Choose one)	1= There is no difference<br>2= At the clinic I would go to learn my status, but in this research I am helping researchers know how to conduct HIV research with adolescents

		3= At the clinic you have to pay money to be tested but in this study it is free to be tested<br>4= I don't know
21.	How long will you be in this study? (Choose one)	1= For the duration of 5 years<br>2= I will be asked by the researchers to give blood one year from today<br>3= I will most likely be done with the study after today, but there is a small chance I may be asked to come back for 2 more interviews<br>4= I don't know
Thank you very much for your participation. We appreciate your help in responding to the questions. Kindly ask the research staff anything you do not understand. Do raise your hand for assistance from the research staff to exit.		
